# Intranasal delivery bypasses the blood-brain barrier to target therapeutic agents to the central nervous system and treat neurodegenerative disease

**DOI:** 10.1186/1471-2202-9-S3-S5

**Published:** 2008-12-10

**Authors:** Leah R Hanson, William H Frey

**Affiliations:** 1 Alzheimer's Research Center at Regions Hospital, HealthPartners Research Foundation, 640 Jackson Street, St. Paul, Minnesota 55101, USA; 2 Department of Pharmaceutics, University of Minnesota, 308 Harvard Street S.E., Minneapolis, Minnesota 55455, USA

## Abstract

Intranasal delivery provides a practical, non-invasive method of bypassing the blood-brain barrier (BBB) to deliver therapeutic agents to the brain and spinal cord. This technology allows drugs that do not cross the BBB to be delivered to the central nervous system within minutes. It also directly delivers drugs that do cross the BBB to the brain, eliminating the need for systemic administration and its potential side effects. This is possible because of the unique connections that the olfactory and trigeminal nerves provide between the brain and external environment. Intranasal delivery does not necessarily require any modification to therapeutic agents. A wide variety of therapeutics, including both small molecules and macromolecules, can be targeted to the olfactory system and connected memory areas affected by Alzheimer's disease. Using the intranasal delivery system, researchers have reversed neurodegeneration and rescued memory in a transgenic mouse model of Alzheimer's disease. Intranasal insulin-like growth factor-I, deferoxamine, and erythropoietin have been shown to protect the brain against stroke in animal models. Intranasal delivery has been used to target the neuroprotective peptide NAP to the brain to treat neurodegeneration. Intranasal fibroblast growth factor-2 and epidermal growth factor have been shown to stimulate neurogenesis in adult animals. Intranasal insulin improves memory, attention, and functioning in patients with Alzheimer's disease or mild cognitive impairment, and even improves memory and mood in normal adult humans. This new method of delivery can revolutionize the treatment of Alzheimer's disease, stroke, and other brain disorders.

## Introduction

The use of intranasal (IN) administration to target therapeutics to the central nervous system (CNS) has many benefits in the treatment of neurologic disorders. The blood-brain barrier (BBB) restricts the use of numerous therapeutic agents that have been developed to treat memory loss and neurodegeneration because it limits CNS penetration, depending on drug size or charge.

Although invasive methods of administration (for instance intracerebroventricular) have been used to overcome the BBB, these methods are not practical for use in humans for several reasons, including convenience, safety, and cost. Direct delivery of therapeutics from the nasal cavity into the CNS (IN delivery) bypasses the BBB and provides an alternative to invasive methods of drug administration [1,2]. Noninvasive IN delivery targets therapeutics to the CNS, reducing systemic exposure and side effects; this can be advantageous for delivery of many CNS therapeutics, including those that can cross the BBB upon systemic administration. CNS therapeutics do not necessarily need to be modified for IN delivery, and delivery of therapeutics to the CNS is rapid, occurring within minutes. The IN delivery method was first developed by Frey in 1989 [3] for targeting neurotrophic factors (for example, nerve growth factor [NGF] and fibroblast growth factor-2) to the CNS.

## Olfactory and trigeminal pathways

Intranasally administered therapeutics reach the CNS via the olfactory and trigeminal neural pathways. Both the olfactory and trigeminal nerves innervate the nasal cavity, providing a direct connection with the CNS. Direct delivery of therapeutics from the nose to the brain was initially attributed to the olfactory pathway [3-6]. More recently, the contribution made by the trigeminal pathway to IN delivery to the CNS has also been recognized, especially to caudal brain regions and the spinal cord [7,8]. Extracellular delivery, rather than axonal transport, is strongly indicated by the short time frame (≤ 10 minutes) observed for IN therapeutics to reach the brain from the nasal mucosa. Possible mechanisms of transport may involve bulk flow and diffusion within perineuronal channels, perivascular spaces, or lymphatic channels directly connected to brain tissue or cerebrospinal fluid [9].

## Intranasal delivery targets therapeutics to the central nervous system

IN delivery has been used to target a wide variety of therapeutics to the CNS. For example, the following classes of therapeutics have successfully been intranasally delivered to the CNS: neurotrophins (NGF [5] and insulin-like growth factor [IGF]-1 [7]); neuropeptides (hypocretin-1 [10] and exendin [11]); cytokines (interferon β-1b [8] and erythropoietin [12]); polynucleotides (DNA plasmids [13] and genes [14]); and small molecules (chemotherapeutics [15] and carbamazepine [16]). IN delivery works best for potent therapeutics that are active in the nanomolar range [1]. Even therapeutics that are substrates for the P-glycoprotein efflux transporter, which is known to operate in the nasal epithelium, have been reported to reach the CNS in effective concentrations [17].

## Intranasal neurotrophins for the treatment of neurodegeneration

Although the ability of neurotrophins to protect and promote the growth of new neurons makes them desirable candidates for the treatment of neurodegenerative disease, the inability of neurotrophins to cross the BBB efficiently, and the impracticality of invasive injection methods, have prevented their use. Growing evidence suggests that IN administration of neurotrophins provides a noninvasive way to target neurotrophins to the CNS to treat neurodegeneration. In a mouse model of Alzheimer's disease, IN administered NGF both reduced neurodegeneration and improved performance in memory tasks [18,19]. In a rat model of stroke, IN IGF-I reduced brain damage and neurologic deficits [20]. In addition, cerebral neurogenesis was induced in the subventricular zone of adult mice after IN administration of FGF-2 [21]. IN activity dependent neurotrophic factor, as well as its active peptide fragment NAP, have been shown to reduce neurodegeneration, tau pathology, amyloid accumulation, and memory loss in mouse models of Alzheimer's disease [22-25]. IN NAP is now being tested in phase II clinical trials as a potential treatment for Alzheimer's disease and mild cognitive impairment.

### Memory is improved by intranasal insulin treatment

IN insulin improves memory in normal adults and patients with Alzheimer's disease without altering blood glucose. Energy metabolism in the CNS is dependent upon glucose uptake and is regulated by insulin in key brain regions. It has long been known that glucose uptake and utilization are deficient in patients with Alzheimer's disease [26]. Recently, the gene expression levels of insulin, IGF-1, and their receptors were shown to be markedly reduced in the brains of patients with Alzheimer's disease [27]. Consequently, ability to deliver insulin to the CNS without altering blood glucose could provide an effective means to improve glucose uptake and utilization, and reduce cognitive deficits in patients with memory disorders.

Using the IN delivery method to target insulin to the CNS originally developed by Frey [28], Born and coworkers [29] demonstrated that cerebrospinal fluid insulin levels significantly increased after treatment of normal adults with insulin, with no change in blood levels of insulin. In normal adults, IN treatment with insulin for 8 weeks improved memory (delayed recall of words) and mood at doses that did not alter blood levels of insulin or glucose [30]. In addition, IN administration of a rapidly acting form of insulin that forms hexamers and is more rapidly absorbed, insulin aspart, improved memory in normal adults significantly more than did regular insulin [31]. In Alzheimer's patients, a single IN treatment acutely improved verbal memory (total story recall and total word list recall) at doses that did not alter blood levels of insulin or glucose [32]. The benefit of IN insulin treatment was seen primarily for Alzheimer's patients without the apolipoprotein E ε4 allele. Longer treatment with IN insulin (21 days) enhanced memory, attention, and functioning compared with placebo in patients with either early stage Alzheimer's disease or mild cognitive impairment [33,34].

## Conclusion

IN delivery is a noninvasive method that bypasses the BBB and targets drugs to the CNS, reducing systemic exposure and side effects. This novel method has already been used successfully to improve memory in both normal adults and patients with Alzheimer's disease. IN delivery could revolutionize the way we treat Alzheimer's disease and other neurodegenerative disorders.

## List of abbreviations used

BBB: blood-brain barrier; CNS: central nervous system; IN: intranasal; NGF: nerve growth factor.

## Competing interests

WHF holds intellectual property related to IN delivery of therapeutics. LRH and WHF are inventors on patents related to IN delivery of therapeutics.
